# Personalized skin health management and nutrition strategies: a comprehensive study based on genetic polymorphisms and dietary factors

**DOI:** 10.3389/fgene.2025.1624960

**Published:** 2025-09-15

**Authors:** Jitao Yang

**Affiliations:** School of Information Science, Beijing Language and Culture University, Beijing, China

**Keywords:** genetic polymorphism, personalized skincare, personalized nutrition, genetic testing, anti-aging

## Abstract

Genetic polymorphism significantly affects an individual’s skin health through various biological pathways such as sensitivity to ultraviolet radiation, antioxidant capacity, inflammatory response, skin barrier function, and natural aging processes. For example, the variation of *MC1R* gene is associated with red hair and low skin pigmentation, increasing sensitivity to UV radiation, which may accelerate the process of photoaging, such as skin sagging, wrinkles, and pigmentation. Therefore, genetic polymorphism is an important factor in the development of personalized skin health management strategies, which helps to better understand the mechanisms of skin problems and provides theoretical basis for scientific skincare. There is a close relationship between diet, skin health, and skin aging. Many basic and clinical studies have confirmed that diet is the main way for humans to obtain the nutrients needed by the body. Adjusting dietary structure and supplementing specific dietary nutrients can have the effect of delaying skin aging. For example, vitamin C is a powerful water-soluble antioxidant that is crucial for collagen biosynthesis. It can directly promote the expression of collagen genes and eliminate intracellular reactive oxygen species, prevent lipid peroxidation, and delay skin aging. Additionally, *SLC23A1* gene encodes antibody transporters, participate in the balance and circulation of vitamin C in the body, and affect the level of vitamin C in the blood. Therefore, in this paper, we integrate multidimensional data including skin genetic testing data, nutrition genetic testing data, dietary and lifestyle questionnaires for data analysis, so that to provide customized nutrition and skincare solutions for each individual. It is expected that combining various omics data and offering personalized solutions will become one of the primary approaches in the field of skin care.

## 1 Introduction

Nowadays, there is a clearer and more accurate understanding of “Precision skincare”. Precision skincare is based on skin science, deeply integrating multidisciplinary theoretical knowledge such as skin pathology and molecular biology. It uses various technical methods such as genomics, metabolomics, and nutrition to explore the specific mechanisms of skin problems. Based on the skin characteristics and problems of different populations, it uses clear molecular targets and functional ingredients, and utilizes emerging technologies and data-driven statistical methods to design personalized cosmetic formulas (for external use), skin health supplements (for oral use), and targeted medical beauty plans. Precision skincare is a scientific and personalized way of skincare, covering “precise identification, precise evidence-based, and precise delivery” to achieve full process management of scientific skincare.

## 2 Skin health

### 2.1 The structure and function of skin

As the second largest organ in the human body, the skin is distributed throughout the body and is mainly composed of water, protein, fat, and minerals. The skin can be divided into three layers from the outside to the inside: epidermal layer, dermis layer, and subcutaneous tissue.

The epidermal layer is divided from the outer to the inner into the stratum corneum, granular layer, spinous layer, and basal layer. The stratum corneum is generally covered with 15–20 layers of corneocytes, which help the skin resist some of the damage caused by ultraviolet rays. The thickness of the stratum corneum not only affect the appearance of the skin, but also is directly related to the skin’s barrier function, sensitivity, nutrient absorption, and the formation of problem skin. Thin stratum corneum can cause skin sensitivity, while thick stratum corneum can hinder the absorption of skincare products and weaken their effectiveness. The granular layer is located below the stratum corneum and consists of two to four layers of polygonal cells. It has almost no activity, but can refract light and reduce UV damage. The spinous layer is composed of 4-8 layers of polygonal cells, which are full of activity and provide nutrition with tissue fluid in the gaps. The deepest layer of the epidermis is the basal layer, which is composed of basal cells and melanin. The content of melanin directly determines the brightness of skin color.

The dermis layer is located beneath the epidermis, with a thickness 7–10 times that of the epidermal layer. It is composed of dense connective tissue, rich in collagen fibers, elastic fibers, matrix, and cellular components, which maintain the structure and elasticity of the skin. The dermis contains abundant capillaries and lymphatic vessels, free nerve endings, and cystic nerve bodies, which collectively participate in sensation, temperature regulation, and overall skin health.

Subcutaneous tissue, also known as subcutaneous fat layer, is located beneath the dermis and is composed of fat lobules and loose connective tissue. It plays a role in insulation and protection, and is also a place for energy storage.

Due to the waterproof and barrier protective effects of the epidermis, daily skincare products can only act on the epidermis. Oral administration of beneficial nutrients such as vitamins, minerals, and antioxidants can penetrate into various layers of the skin, improving skin health from the inside out and achieving longer lasting skincare effects.

### 2.2 Mechanisms and manifestations of skin aging

Skin aging can be divided into endogenous aging and exogenous aging. Endogenous aging is influenced by genetic, endocrine, and other factors, including: decreased proliferation ability of skin derived cells; Reduced synthesis of dermal matrix; The expression of enzymes that degrade collagen matrix increases. In addition, with the oxidative damage and accumulation of glycation products in cells, it can also cause the adhesion between fiber cells, the disintegration of the network structure, and the loss of support for the skin (Gerasymchuk et al., 2020). A study compared the proliferation ability of elderly skin derived cells and young skin derived cells, and found that the division and proliferation ability of skin cells decrease with age, manifested as growth arrest and cell aging. The rate of collagen biosynthesis in the skin of elderly people decreases, causing cells to transition from the matrix production phase in youth to the matrix degradation phase in old age, resulting in a decrease and breakdown of collagen. The final manifestation is atrophy of the epidermal layer, loss of epidermal moisture, decreased barrier function, reduced production and degradation of collagen and elastin fibers in the dermis, loss of skin elasticity, and wrinkles.

Exogenous aging is the result of a combination of environmental factors such as ultraviolet radiation, smoking, air pollution, etc. Among them, skin photoaging caused by ultraviolet radiation has the most significant impact on skin aging. Ultraviolet radiation can penetrate the skin and damage DNA, causing collagen and elastin degradation, resulting in skin loss of elasticity, roughness, dryness, pigmentation, and deep wrinkling (Schweitzer and Maibach, 2014). External pollutants, tobacco smoke, and other substances can cause oxidative stress on the skin, producing a large amount of free radicals, leading to dysfunction of skin cells and tissue damage. External stimuli such as chemicals, dust, microorganisms, etc. Can cause skin inflammation. Long term chronic inflammation can accelerate skin aging, make the skin more sensitive to external stimuli, and disrupt skin stability.

## 3 Genes and skin health

Genetic polymorphism significantly affects an individual’s skin health through various biological pathways such as sensitivity to ultraviolet radiation, antioxidant capacity, inflammatory response, skin barrier function, and natural aging processes. Specific genetic variations may increase the risk of skin cancer, affect the skin’s resistance to environmental factors, and enhance its ability to repair and regenerate. Therefore, genetic polymorphism is an important factor in the development of personalized skin health management strategies, which helps to better understand the mechanisms of skin problems and provides theoretical basis for scientific skincare.

### 3.1 Genes and skin photoaging

Skin photoaging is a complex biological process mainly caused by long-term ultraviolet (UV) radiation, involving multiple genes and their polymorphisms. These gene polymorphisms can affect the skin’s sensitivity to UV radiation, antioxidant defense ability, inflammatory response, and skin repair mechanisms. For example, the rs4268748 locus of the MC1R gene is significantly associated with skin aging characteristics. The variation of MC1R gene is associated with red hair and low skin pigmentation, increasing sensitivity to UV radiation, which may accelerate the process of photoaging, such as skin sagging, wrinkles, and pigmentation. In addition, variations in the MC1R gene may also affect an individual’s ability to repair DNA damage caused by ultraviolet radiation, thereby affecting the process of skin aging. The rs12203592 locus of the IRF4 gene is associated with reduced skin pigmentation, skin aging, and increased risk of skin cancer. The rs185146 polymorphism of SLC45A2 gene is associated with skin aging characteristics, affecting the generation and distribution of skin pigments (Markiewicz and Idowu, 2022; Markiewicz and Idowu, 2018).

Individuals carrying the above gene mutations are recommended to supplement with antioxidant functional foods or active substances, sunscreen products, etc. to reduce UV radiation damage to the skin and delay the aging process of the skin. Genetic polymorphism plays a crucial role in skin photoaging, providing important biological basis for skin health management and disease prevention.

### 3.2 Genes and skin pigmentation

Skin pigmentation refers to the phenomenon of darkening of skin or mucous membrane color, skin pigmentation disorders are usually associated with abnormal increase of melanin. The melanin produced by melanocytes is the main determinant of skin color, and changes in its quantity and distribution directly affect the color and glossiness of the skin. Skin pigmentation can be caused by various factors, including ultraviolet radiation, endocrine disorders, genetic factors, drug side effects, and post inflammatory changes. Among them, genetic factors mainly refer to the fact that gene polymorphism can affect the synthesis, transportation, degradation of melanin, as well as the function and distribution of melanocytes through various biological pathways.

Influence melanin synthesis or pigment proportion: TYR (tyrosinase) gene plays a key role in the biosynthesis of melanin. Genetic variation can change the activities of these enzymes, thus, tyrosinase encoded by TYR gene and tyrosinase related protein one encoded by TYRP1 gene are key enzymes in the melanin synthesis pathway, affecting the production and type of melanin, such as the proportion of brown melanin and yellow red pigment, their mutations can lead to different degrees of pigmentation abnormalities (Saternus et al., 2015). Melanocortin receptor one encoded by MC1R gene is involved in regulating the proliferation and differentiation of melanocytes. Genetic variation may affect the function of this receptor, and then affect the production of melanin. The signal protein encoded by ASIP gene is an antagonist of MC1R, gene mutation will change the synthesis ratio of brown melanin and yellow red pigment, and then affect skin color (Sinnott-Armstrong et al., 2021). MITF gene is a major regulator in the synthesis of melanocytes and their pigment cells, and its mutation can affect the development and function of melanocytes (Bajpai V. et al., 2023). IRF4 gene is involved in the regulation of melanin synthesis and is associated with skin tanning response (Palstra and Kayser, 2015). In addition, genetic variations in some genes such as EXOC2 may affect the synthesis of vitamin D in the skin and indirectly affect skin pigmentation (Hamer et al., 2018).

Influence on melanosome maturation and trafficking (Law et al., 2017): OCA2 gene is involved in regulating melanosome maturation and tyrosinase trafficking, and genetic variation may affect the function and stability of melanosomes. HERC2 gene affects the cell cycle and DNA damage response by regulating the activity of ubiquitin protein ligase, and interacts with OCA2 gene to regulate the process of skin pigmentation. SLC45A2 gene is responsible for transporting pre melanin protein into melanosomes, and its variation is related to the regulation of pigmentation. DCT gene is involved in the maturation process of melanin, and its polymorphism is related to the regulation of pigmentation, affecting skin pigmentation and photoprotection.

Influence on melanocyte degradation and cell function (Endo et al., 2018): GGT7 gene polymorphism may affect the process of autophagy, and then affect the degradation of melanosomes in melanocytes. RALY gene polymorphisms may affect the structure and function of melanocytes, including melanosome biosynthesis and trafficking. KITLG gene polymorphism is associated with the development and migration of melanocytes.

Influence on pigmentation: BNC2 gene is related to the formation of facial pigment spots. PGC-1 β encoded by PPARGC1B gene is related to energy metabolism and pigmentation, and its variation may affect the skin response to sunlight, which is related to the formation of color spots (Shoag et al., 2014). The MFSD12 gene is involved in the transport and distribution of melanocytes and is associated with skin pigmentation (Adelmann et al., 2020). CDKN2A gene encodes a cell cycle inhibitory protein, and its variation may affect the proliferation and apoptosis of melanocytes, which is related to the formation of color spots (Ibarrola-Villava et al., 2010). FAM20C gene regulates the skin response to UV light and is associated with skin pigmentation, GPR143 gene polymorphism is associated with the color of iris and the formation of skin patches (Jonnalagadda et al., 2019; Bajpai VK. et al., 2023).

### 3.3 Genes and skin elasticity

The formation of wrinkles is a natural manifestation of the decline of skin elasticity with aging, and this process is significantly affected by genetic and environmental factors. Genetic factors show individual differences in the rate of skin aging and the degree of wrinkles by affecting the structure and function of the skin and the response to environmental pressure. Some genes are directly involved in the synthesis and metabolism of collagen and elastin, and are essential for the structural maintenance and repair ability of skin (Park et al., 2021; Liu N. et al., 2019; Liu Y. et al., 2019). For example, the COL1A1 gene and COL1A2 gene encode the α1 and α2 chains of type I collagen (the most abundant collagen type in the skin), and polymorphisms in related gene loci affect skin structure and elasticity. COL3A1 gene encodes the α1 chain of type III collagen, which affects skin elasticity and wound healing. COL5A1 gene and COL5A2 gene encode α1 and α2 chains of type V collagen, which are involved in skin repair and regeneration. COL17A1 gene (also known as BP180 gene or BPAG2 gene) encodes type XVII collagen, which is an important component of anchoring fibers and contributes to the connection between epidermis and dermis. ELN gene encodes elastin, which is the main component of elastic fibers and is essential for skin elasticity (Hoang et al., 2024). FBN1 gene encodes fibronectin, which is involved in the formation of extracellular matrix and cell-cell adhesion, affecting the assembly of elastin fibers and the maintenance of skin structure (Gao et al., 2017). EGFR gene encodes epidermal growth factor receptor, which is involved in regulating cell proliferation and differentiation and affecting collagen synthesis. The above gene variations will directly affect the structure and function of skin, causing premature aging such as decreased skin elasticity and deepened wrinkles (Romana-Souza et al., 2019).

Some genes also affect the degradation of collagen and elastin and the skin barrier function, such as MMP1, MMP3, MMP9, MMP12, and MMP16 (Cui et al., 2018). These genes encode matrix metalloproteinases (MMPs), which are involved in the degradation of collagen and elastin and the remodeling of the extracellular matrix, affecting the repair of skin damage. Mutations in related gene loci can cause collagen degradation, leading to skin sagging and wrinkles (Re et al., 2020). The COL17A1 gene is related to the structure and integrity of the skin, affecting the skin’s repair of external damage (such as ultraviolet rays), and indirectly affecting the formation of spots (Endo et al., 2018). The LOXL1 and LOXL2 genes encode lysyl oxidases, which participate in the cross-linking process of elastin and collagen and affect the structural integrity of the skin. The SOD3 gene encodes superoxide dismutase 3, an antioxidant enzyme that helps protect the skin from oxidative stress damage and indirectly affects the synthesis and stability of collagen (Chan et al., 2019; Seo et al., 2022). Although the TGF- β 1 gene is not directly involved in the synthesis of collagen, the transforming growth factor- β 1 it encodes plays a key role in regulating the synthesis of collagen. Keratin 5 and 14, encoded by the KRT5 and KRT14 genes, are structural proteins of epidermal cells and are related to the skin barrier function (Moon et al., 2018; Chan et al., 2019).

### 3.4 Genes and skin sensitivity

Some improper skin care habits such as excessive cleansing, frequent use of skin care products containing irritating ingredients, and adverse environmental stimuli such as dryness, cold or ultraviolet radiation will reduce the skin’s barrier function, causing external irritants to penetrate the skin more easily, easily causing inflammatory reactions and sensitive symptoms, manifested as erythema, itching, stinging or burning sensation.

Genetic defects in proteins related to the skin barrier can cause skin barrier dysfunction. For example, the filament-related protein encoded by the FLG gene is an important structural protein in the skin barrier. It is produced in the stratum corneum of the epidermis and has the function of cross-linking keratin fibers. After sufficient cross-linking, it forms a corneocyte membrane. The main function of the corneocyte membrane is to form a barrier in the outermost layer of the skin to prevent water loss and prevent the invasion of external allergens and infectious factors. Mutations in the FLG gene-related loci can cause skin allergy symptoms (Drislane and Irvine, 2020). The SPRR3 gene encodes a corneocyte envelope precursor protein. Mutations in the related loci can cause corneocyte envelope defects, which are thinner than normal envelopes, resulting in a decrease in extracellular lipids and a weakening of the adhesion between the stratum corneum, which manifests as dry and sensitive skin (Løset et al., 2019).

Gene mutations related to proteases or protease inhibitors can also cause defects in skin barrier function. For example, human tissue kallikrein encoded by the KLK7 gene and the KLK5 gene is the main protease in the process of epidermal desquamation. Mutations in related loci can cause skin barrier disorders (Morizane et al., 2012). The SPINK5 gene encodes a serine protease inhibitor (LEKTI), which regulates the formation of epithelial cells and the proteolysis during the terminal differentiation of keratinocytes, as well as the normal epithelial generation process. Mutations in related loci can cause changes in the structure, function, and concentration of LEKTI, ultimately leading to skin barrier abnormalities (Zhao et al., 2012). Mutations in the TMEM79 gene (also known as the MATT gene) can cause a decrease in the level of the protein mattrin it encodes, hinder the secretion of the contents of the lamellar bodies, and damage the transmission of the components of the lamellar bodies, causing skin itching and skin lesions (Emrick et al., 2018).

### 3.5 Genes and skin repair

The skin anti-aging repair mechanism refers to a series of complex physiological processes in which the skin, through its own physiological regulation or external intervention, combats the structural and functional decline caused by endogenous aging (natural aging) and exogenous aging (such as ultraviolet rays, pollution, and bad living habits), thus maintaining or restoring a youthful state. The repair mechanism of skin anti-aging involves multiple aspects, including cell renewal, molecular signaling, maintenance of the extracellular matrix, and protection against oxidative stress.

In the process of minor skin impairment repair, different types of skin cells, such as epidermal stem cells, keratinocytes, fibroblasts, etc., will migrate to the damaged area after the damage occurs to promote damage healing. Mutations in the FSCN1 gene-related loci are related to adhesion between injured skin cells, which will affect cell migration and wound closure (Horsley, 2020).

Skin cells neutralize reactive oxygen species, eliminate free radicals and toxic substances in the body, and resist oxidative damage within the body and environment by producing antioxidant enzymes. For example, the glutathione peroxidase (GPx) gene encodes GPx, which can degrade hydrogen peroxide and other organic peroxides, protecting cells from oxidative damage. The heme oxygenase (HO-1) gene encodes HO-1, a stress protein with antioxidant and anti-inflammatory properties that can protect skin cells from oxidative stress damage. The NAD (P) H quinone oxidoreductase 1 (NQO1) gene encodes NQO1, which can catalyze the reduction of quinone substances, reduce their production of reactive oxygen species (ROS), and protect cells from oxidative stress. Mutations in the above gene related loci can reduce antioxidant enzyme activity and affect skin health (Chen et al., 2021).

Skin cells can secrete anti-inflammatory molecules, reduce inflammatory responses, and prevent inflammatory damage during skin aging. A French Atopic Dermatitis Cohort study shows that the genes FLG, KLK seven and SPINK5 are involved in maintaining the skin barrier function and play an important role in maintaining the skin barrier and anti-inflammation (Hubiche et al., 2007). Th2 immune response-related genes, such as the IL4 gene and IL13 gene, participate in the type 2 T helper lymphocyte (Th2) signaling pathway, affect the Th2 immune response, and are related to the skin’s anti-inflammatory mechanism (Zhu, 2015; Bao and Reinhardt, 2015). The DDX5 gene and IL-17D gene affect IL-36R-mediated inflammatory responses (Ni et al., 2022).

Environmental factors such as ultraviolet radiation can cause DNA damage, and skin cells have the ability to repair DNA damage. The SIRT1 gene promotes DNA repair by regulating the activity of multiple DNA repair proteins, and mutations in its related gene loci can affect the skin’s ability to repair DNA damage. Genes involved in DNA damage repair mechanisms such as base excision repair (BER), nucleotide excision repair (ENR), mismatch repair (MMR), double-strand break repair, and translesion synthesis (TLS), such as POL δ / ϵ / κ , PCNA, RFC, XPC, and other gene polymorphisms can affect the efficiency of DNA repair, thereby affecting the skin’s ability to repair damage (Dong et al., 2017).

## 4 Nutrition and skin health

There is a close relationship between diet, skin health, and skin aging. Many basic and clinical studies have confirmed that diet is the main way for humans to obtain the nutrients needed by the body. Adjusting dietary structure and supplementing specific dietary nutrients can have the effect of delaying skin aging. People’s interest and willingness to fight aging through nutrition and dietary supplements are becoming increasingly strong.

### 4.1 Basic nutrients

Vitamins play an important role in delaying skin aging and improving skin appearance. Many vitamins have antioxidant properties that can clear reactive oxygen species and reduce oxidative damage to cells. For example:

• Vitamin A and its derivatives are one of the most common nutrients for delaying skin aging. Clinical studies have shown that oral supplementation of vitamin A and its derivatives can alleviate skin photoaging, promote fibroblast activity, reduce metalloproteinase activity, promote collagen synthesis, and reduce collagen breakdown. Retinol is a common form of vitamin A supplementation, which can significantly improve the dermal microenvironment, stimulate skin cells to produce collagen, fibronectin, elastin, and promote dermal angiogenesis (Shao et al., 2017). A study has shown that applying 3% retinol for six consecutive weeks significantly improves subjects’ skin in terms of fine lines, wrinkles, pore size, sagging, pigmentation, clarity, brightness, and overall light damage (Sadick et al., 2019).

• Vitamin C is a powerful water-soluble antioxidant that is crucial for collagen biosynthesis. It can directly promote the expression of collagen genes and eliminate intracellular reactive oxygen species, prevent lipid peroxidation, and delay skin aging. Oral supplementation of vitamin C can prevent dry skin, reduce wrinkle formation, and promote wound healing (Pullar et al., 2017; Boyce et al., 2002; Bocheva et al., 2022). A study has shown that applying cosmetics containing 20% vitamin C for eight consecutive weeks significantly improves the color, elasticity, and radiance of the subjects’ skin (Milani and Sparavigna, 2018).

• Vitamin E is a free radical scavenger that acts as an antioxidant in the body, stabilizing cell membranes and protecting membrane proteins. Vitamin E can protect the skin under oxidative stress, prevent collagen cross-linking, significantly enhance the skin’s own barrier protection function, make the skin have higher elasticity, thicker dermis layer and thinner epidermis layer. In addition, it can increase skin moisture content, improve skin moisturizing performance, enhance skin smoothness, and have a protective effect on ultraviolet radiation. The combined application of vitamin E and C can help activate vitamin E and protect the skin from chemical and UV induced irritation and damage by inhibiting lipid peroxidation in the skin (Keen and Hassan, 2016; Wu et al., 2013)

Minerals in the body mainly support the immune system and promote the production of hormones and enzymes, which are important factors affecting enzyme activity in skin metabolism (Haftek et al., 2022). For example:

• Magnesium is the main component in controlling carbohydrate, protein, and energy metabolism processes, which can maintain the functional and structural integrity of cells, play an anti fatigue and anti stress role, and promote the repair of epidermal sebum membrane (Yoshino et al., 2024).

• Zinc is an essential element for synthesizing nucleic acids and is a necessary component for wound healing and new cell growth. Zinc can make the skin smooth, delicate, and elastic. When zinc is deficient, growth and development may be delayed, the skin may become rough, and pigmentation may increase. Zinc oxide and zinc carbonate are widely used in baby urine rash cream, relieving cream and lotion, with the functions of convergence, mild sterilization and relieving skin pressure (Schwartz et al., 2005).

• Copper can eliminate free radicals in the body, assist vitamin C in synthesizing elastin, and also produce disulfide crosslinks in the stratum corneum, promoting hair growth. Copper can participate in the healing and regeneration of epidermal molecules, as well as participate in anti-inflammatory effects. Copper plays an important role in the formation of tyrosinase, which activates melanocytes to produce melanin. Therefore, copper has the ability to naturally darken the skin and help maintain this color (Borkow, 2014).

• Silicon plays an important role in the formation of collagen and connective tissue, hair, skin, and nails, and has a certain anti-aging effect (Bains et al., 2023).

• Selenium is a component of glutathione peroxidase in human red blood cells, which can protect cell membranes, eliminate harmful free radicals, and is a strong antioxidant. Can maintain tissue elasticity and delay aging. Relieve heavy metal poisoning such as arsenic, mercury, and lead, inhibit dandruff, and moisturize the skin (Bjørklund et al., 2022).

• Iron can provide oxygen to skin cells, promote cellular respiration, and make the skin moist and elastic. Iron deficiency can lead to anemia and pale complexion. Iron can also promote fatigue relief and promote skin health (Wright et al., 2014).

• Iodine can stimulate the secretion of thyroid hormone, which is closely related to normal hair growth and can increase hair shine while preventing hair splitting (Greenwald et al., 2017).

• Germanium is an antioxidant that can prevent lipid peroxidation, maintain skin elasticity, delay the appearance of wrinkles, whiten and beautify the skin. It has a lightening effect on abnormal pigmentation caused by childbirth or sunlight, and can also assist in the treatment of acne, eczema, and underarm odor, effectively preventing skin and blood cancer (Matsumoto et al., 2016; Takeda et al., 2019).

Fatty acids are an important component of the skin, closely related to the function of the epidermal barrier, membrane structure, homeostasis of the internal environment, and damage repair. Skin aging is also accompanied by a decrease in lipid content (Pappas et al., 2013). The symptoms of fatty acid deficiency include dry epidermis, peeling, loose skin, skin inflammation, sebaceous gland obstruction, susceptibility to irritation, and slow healing. Studies have shown that polyunsaturated fatty acids, including omega-3 and omega-6 fatty acids, have a positive effect on skin moisturizing and barrier function. Omega-3 fatty acids can promote skin collagen synthesis, inhibit skin reactive oxygen species, lipid peroxidation, protein carbonylation, and MMP-8 expression, and slow down skin aging induced by chronic psychological stress (Romana-Souza and Monte-Alto-Costa, 2019). Palmolive oil can alleviate UV induced skin photoaging in mice, improve skin thickness, skin barrier function, and wrinkle related evaluation indicators, and reduce the expression of MMP-3 (Park et al., 2019).

### 4.2 Active functional ingredients

#### 4.2.1 Polyphenols

Polyphenols are widely present in the plant world and are organic compounds containing two or more hydroxyl groups attached to aromatic rings (Tsao, 2010). According to their chemical structure, phenolic compounds can be divided into five categories: flavonoids, phenolic acids, tannins, stilbenes, and plant polyphenols. They are important substances for maintaining skin function and have anti-inflammatory, antibacterial, antifungal, antiviral, anti allergic, anticancer, and anticoagulant properties that help moisturize, smooth, soften, soothe, and converge the skin (Shahidi and Ambigaipalan, 2015; Merck et al., 2008). Polyphenols can inhibit the activity of collagenase, elastase, and hyaluronidase in the skin, which catalyze the hydrolysis of collagen, elastin fibers, and hyaluronic acid (Nichols and Katiyar, 2010). Polyphenols can also soothe irritation and reduce skin redness, accelerate natural regeneration of the epidermis, stabilize capillaries, improve skin microcirculation and elasticity, and protect the skin from environmental factors (including ultraviolet radiation) (Zillich et al., 2015). Common polyphenolic compounds include silymarin, genistein, curcumin, resveratrol, tea polyphenols (epicatechin, epicatechin gallate, epigallocatechin gallate), psoralen, flavonoids, phenolic acids, tannins, stilbene, and diphenylmethane (Chuarienthong et al., 2010).

#### 4.2.2 Carotenoids

Carotenoids can prevent aging, stimulate fibroblasts to produce collagen and elastin, inhibit MMP activity, and have anti-inflammatory and UV filtering effects (Eldahshan and Singab, 2013; Davinelli et al., 2018; Mezzomo and Ferreira, 2016; Darvin et al., 2011). Carotenoids can improve the elasticity, moisture, and texture of the skin, enhance barrier integrity, whiten the skin, and delay photoaging. Common carotenoid compounds include beta carotene, lycopene, astaxanthin, lutein, zeaxanthin, cryptoxanthin, fucoxanthin, etc (Tominaga et al., 2012; Yoon et al., 2014; Juturu et al., 2016; Schwartz et al., 2016; Meephansan et al., 2017).

#### 4.2.3 Polysaccharides

Polysaccharides have many effects such as improving immune function, anti-tumor, antiviral, anti glycation, antioxidant, etc. Polysaccharides can enhance skin antioxidant enzyme activity, eliminate reactive oxygen species, reduce oxidative damage, inhibit cell apoptosis. Polysaccharides inhibit collagen degradation by suppressing the expression of enzymes such as MMP-1 and MMP-9, maintain a stable collagen ratio, repair skin structure, and maintain skin moisture content Common polysaccharide compounds include Spirulina polysaccharide, Sargassum polysaccharide, Tremella fuciformis polysaccharide (Wen et al., 2016), Ginseng polysaccharide, Bamboo leaf polysaccharide, Ganoderma lucidum polysaccharide, Houttuynia cordata polysaccharide, Flammulina velutipes polysaccharide, Cordyceps sinensis polysaccharide, etc (Wang et al., 2018; Ye et al., 2018). Hyaluronic acid is also a widely used polysaccharide that plays a crucial role in maintaining skin structure. Oral supplementation of hyaluronic acid significantly increased the elasticity and strength of the subjects’ skin, significantly increased skin hydration, and significantly reduced skin roughness and wrinkle depth (Gollner et al., 2017).

#### 4.2.4 Collagen

A series of small molecule peptides produced by the hydrolysis of collagen, with low molecular weight, easy absorption, anti-inflammatory and antioxidant properties, widely used to delay skin aging. Collagen peptides mainly come from animal tissues such as skin, bones, and muscles. As a precursor for collagen synthesis, collagen peptides can: 1) delay skin aging, 2) participate in collagen synthesis, 3) regulate cytokines to promote collagen and hyaluronic acid synthesis, 4) inhibit the activity of proteases including MMP-3 and reduce collagen degradation 5) participate in clearing reactive oxygen species within cells, and 6) reduce oxidative damage and inflammatory reactions within cells (Muzumdar and Ferenczi, 2021).

#### 4.2.5 Other active ingredients

Mulberry root extract, peony root extract, licorice extract, etc., help with anti-inflammatory effects on the skin; Glutathione, superoxide dismutase, proanthocyanidins, idebenone, coenzyme Q10, fullerenes, myopeptides, ergothionein, peony root extract, dendrobium officinale polyphenols, root bark extract, etc. are beneficial for skin antioxidant properties; Niacinamide, ergotamine, α lipoic acid, etc. help the skin resist glycation (McDaniel et al., 2018; Bae et al., 2019).

Skullcap root extract, carpamos longum extract, hydrolyzed soy protein, etc. help protect DNA telomeres (Jacczak et al., 2021).

Low concentration of small molecule alpha hydroxy acids such as glycolic acid, fermentation products of bifidobacteria, fermentation filtrate of Aspergillus niger, and fermentation filtrate of galactose yeast like bacteria can help correct cell apoptosis and promote cell regeneration (Reilly and Lozano, 2021; Endo et al., 2020).

Niacinamide, prickly fruit oil, Cordyceps sinensis fermentation filtrate, and algae extracts containing ${\text{Ca}}^{2+}$ can promote keratin synthesis (Emmetsberger and Mammone, 2021).

Ceramides, free fatty acids, cholesterol, and other substances can supplement physiological lipids and repair the skin barrier (Choi et al., 2022).

Extracts of *centella asiatica* and peptide active substances such as palmitoyl tripeptide-1, hexapeptide-9, palmitoyl hexapeptide-12, Cordyceps sinensis fermentation filtrate, recombinant collagen, etc. Can promote fibroblast proliferation and increase the secretion of collagen and elastin (Prommaban et al., 2022).

High concentrations of small molecule α hydroxy acids such as glycolic acid, small molecule hyaluronic acid, C-xylose glycosides, etc. Can promote the synthesis of glycosaminoglycans, promote the synthesis of collagen and elastin fibers in the dermis, and supplement the matrix; Extracts of Chrysanthemum morifolium, dipeptidyl butyrylbenzylamide diacetate, acetyl hexapeptide-8, β - propargyl hydroxyprolinyl dimethylaminobutyric acid benzylamine, etc. Can inhibit the contraction of expressive muscles and reduce the production of expressive lines; Caffeine, α - new endorphin, Ginseng fruit extract, etc. Can promote autophagy of skin aging cells (Lim et al., 2020; Li et al., 2018).

Plant sterols such as aloe vera sterols help protect the skin from free radical damage, regulate skin oil secretion, reduce inflammatory reactions, and help delay the skin aging process (Tanaka et al., 2016).

### 4.3 Common active ingredients in cosmetics

Active ingredients are added to cosmetics in order to achieve whitening, sun protection, anti-allergy, anti-acne, anti-aging, moisturizing and other effects.

Peptides, a common functional ingredient in first-line cosmetic brands, have the functions of promoting collagen production, anti-free radical oxidation, anti-inflammatory repair, anti-edema, promoting hair regeneration, whitening, etc. The most common signal peptides in skin care products are: palmitoyl pentapeptide-3, palmitoyl tripeptide-1, palmitoyl hexapeptide, palmitoyl tripeptide-5, hexapeptide-9, myristoyl pentapeptide-11. It can promote the synthesis of matrix proteins, especially collagen, while increasing the production of elastin, hyaluronic acid, glycosaminoglycans and fibronectin. Neurotransmitter inhibitory peptides mainly inhibit the synthesis of SNARE receptors, inhibit the excessive release of catecholamines and acetylcholine in the skin, locally block the nerves from transmitting muscle contraction information, relax the facial muscles, and achieve the purpose of smoothing fine lines. Such as acetyl hexapeptide-8, acetyl octapeptide-3, pentapeptide-3, dipeptide-2. Carrier peptides mainly achieve anti-aging effects by transporting trace elements needed by the skin to the site of action. The most representative of these is copper peptide (also known as “blue copper peptide”) (Skibska and Perlikowska, 2021).

Vitamin A, niacinamide, vitamin E, and vitamin C are often added to cosmetic products. Topical retinols can penetrate the keratinized epidermis and enter the dermis and subcutaneous tissue in small amounts. Epidermal renewal is achieved by promoting the shedding of keratinocytes and stimulating the proliferation of living epidermal cells. It enhances the barrier function of the epidermis, reduces water loss, stimulates fibroblasts, and increases the synthesis of elastin and collagen. Retinol can reduce skin pigmentation by about 60% and inhibit the transport of melanin to epidermal cells. Topical vitamin C can combat UVA-induced oxidative stress and play an important role in protecting against UVA damage. Vitamin C is an essential cofactor for collagen synthesis. It directly stimulates collagen production by activating proline/lysine hydroxylase and promoting collagen mRNA expression. The combined use of vitamin C and vitamin E has a stronger photoprotective effect and can also improve pigmentation. However, topical vitamin products are prone to side effects such as skin redness, desquamation, and skin sensitivity (Michalak et al., 2021).

Human growth factor (HGF) is a cytokine that exists in the body and regulates (promotes or inhibits) the growth and development of different cells in the body. It is a type of compound hormone that controls the growth and differentiation of skin, blood, bone and nerve tissue cells and can be used for anti-aging of the skin. Commonly used growth factors in cosmetics include human epidermal growth factor (hEGF), basic fibroblast growth factor (bFGF), keratinocyte growth factor (KGF), vascular endothelial growth factor (VEGF), etc (Qin et al., 2017).

Acidic substances can change the acidity of the skin surface, remove excess keratin on the skin surface, and promote the normal metabolism of skin cells. They are suitable for various skin problems caused by excessive oil secretion and keratin accumulation. They have the effects of controlling oil, exfoliating, removing acne, shrinking pores, and fading marks. Common acidic substances include fruit acids and salicylic acid (Pandey et al., 2023).

## 5 Personalized skin health solution

### 5.1 Skincare genetic testing

As mentioned above, there are multiple genes in the human body that are associated with skin aging, the polymorphism of gene loci related to collagen synthesis and degradation, skin moisturizing ability, antioxidant ability, and damage repair ability can all affect the anti-aging ability of the skin. Therefore, we developed a skincare genetic testing service, which provides users with six major skin ability testing categaries, including:

• Anti inflammatory and anti allergic: which includes the genetic testing items of: Skin Protection ability, Skin Anti-inflammatory ability;

• Wrinkles reduction: which includes the genetic testing items of: Anti Fat Mass ability, Anti Varicose Vein ability, Anti Stretch Mark ability;

• Acne treatment: which includes the genetic testing items of: Anti acne ability, Skin repair ability;

• Spots Lightening and Whitening: which includes the genetic testing items of: Anti Tanning ability, Anti Sunburn ability, Anti photoaging ability, Anti Sunspot ability;

• Moisturizing: which includes the genetic testing items of: Skin Water Locking ability, Skin Nutrient Absorption ability;

• Skin rejuvenation and anti aging: which includes the genetic testing items of: Antioxidant ability, Anti Wrinkle ability, Anti Glycation ability.

To provide genetic testing services for users, we send a saliva sampling kit to user’s home. The user follows the instructions to spit saliva into a collection tube containing preservation solution, and then mails it to our laboratory. We extract oral cells from the user’s saliva, further extract DNA, and then perform genetic sequencing using next-generation sequencing technology.

Each skin care genetic testing item has its own independent evaluation algorithm, for example, additive allele model was used to quantify the association between the SNPs and anti wrinkle ability (Ng and Chew, 2022).

**FIGURE 1:**
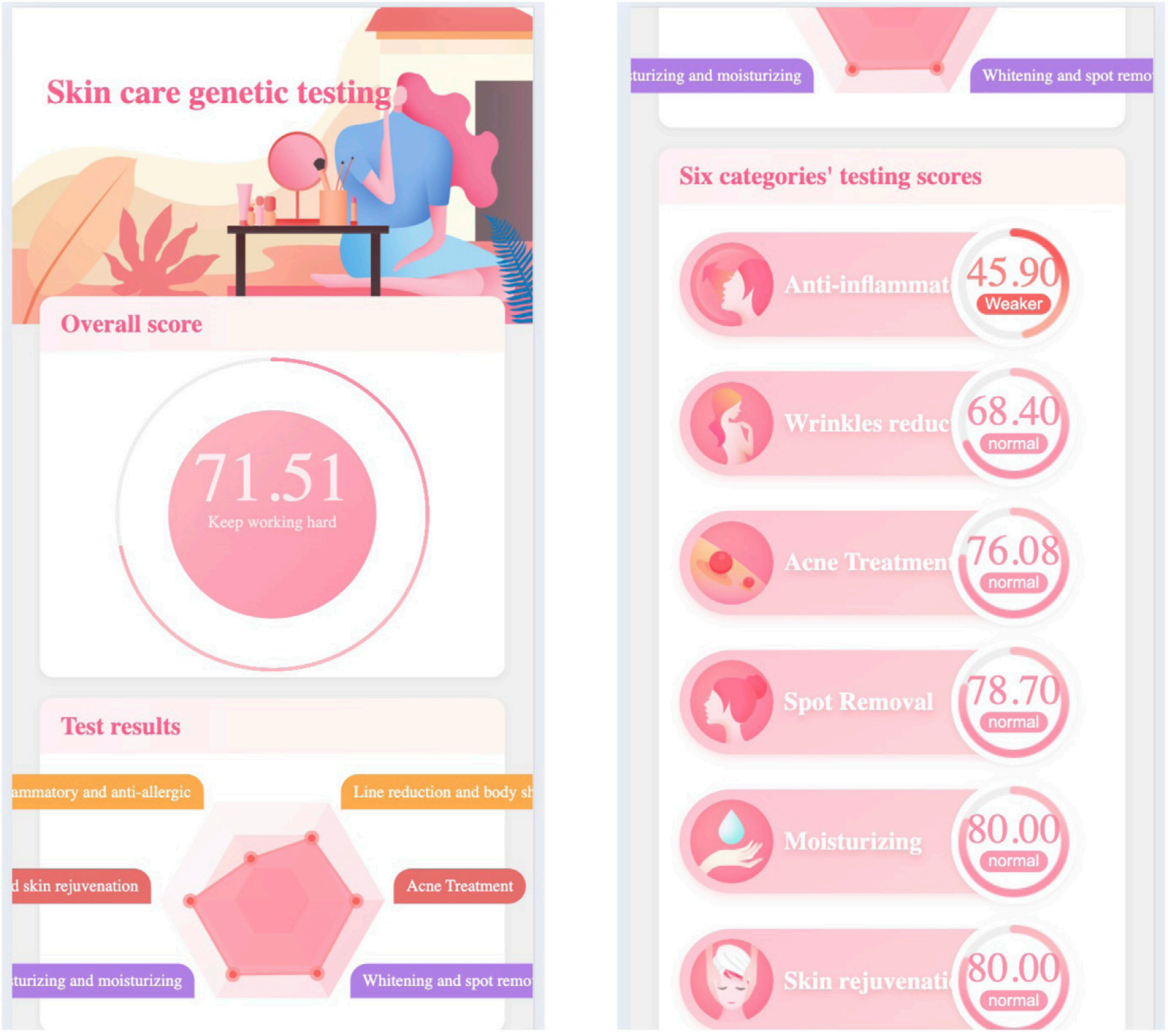
The skincare genetic testing report’s first page.

Figure 1 demonstrates the skincare genetic testing report’s first page, in which.

• The first part gives a overal rating first,

• The second part displays skin ability in six dimensions through radar charts,

• The third part lists skin ability testing scores in six categaries (e.g., anti-inflammatory).

**FIGURE 2:**
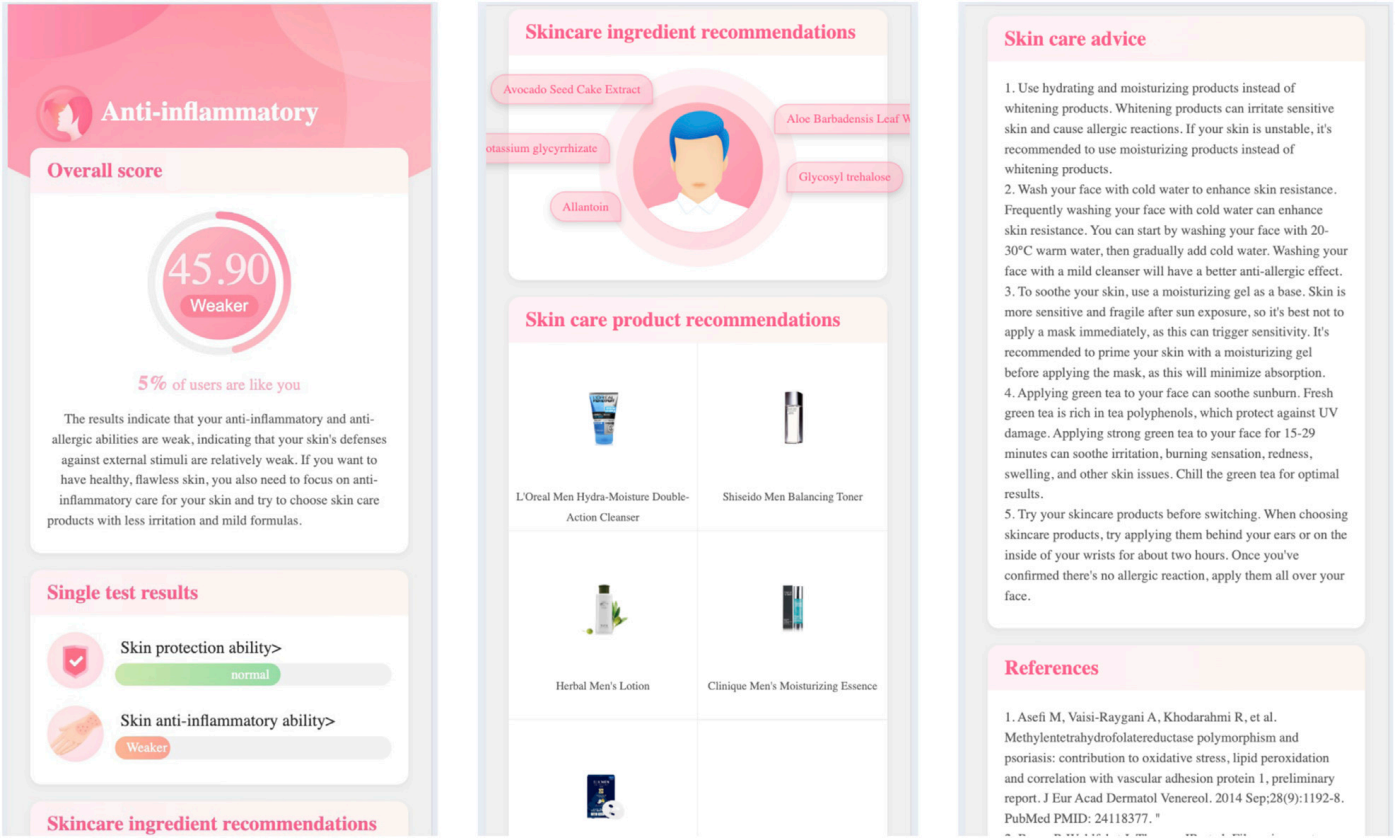
The skincare genetic testing report’s second-level page.

Click the testing categary’s name (e.g., anti-inflammatory), the report page will open the second level page. Figure 2 demonstrates the skincare genetic testing report’s second level page, in which.

• The first module shows the overall ratings, compares with other people, and gives a short explanation of the testing results;

• The second module lists the genetic testing items in the anti-inflammatory categary (i.e., skin protection ability, and skin anti-inflammatory ability);

• The third module recommends the skin care ingredients, e.g., Avocado Seed Cake Extract;

• The fourth module recommends the skin care products;

• The fifth module gives skincare advices;

• The sixth module lists the reference papers.

**FIGURE 3:**
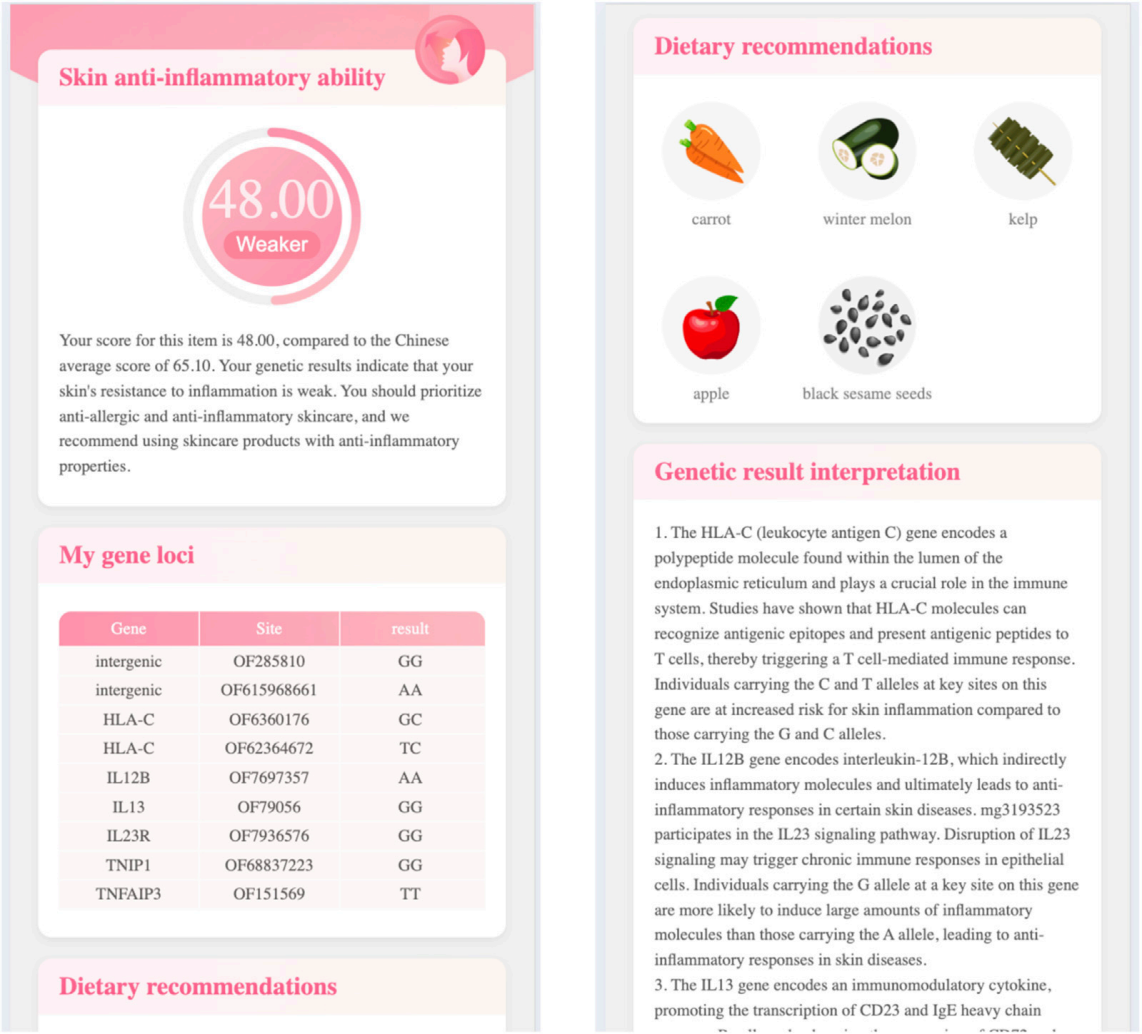
The skincare genetic testing report’s third-level page.

Click the single testing item’s name (e.g., skin anti-inflammatory ability), the report page will open the third level page. Figure 3 demonstrates the skincare genetic testing report’s third level page, in which.

• The first module gives the testing score and explanations;

• The second module lists the tested genes and loci;

• The third module gives dietary advice, which lists the foods includes the ingredients that will help to pomote skin anti-inflammatory ability;

• The fourth module lists the references.

By testing the relevant genes, we can identify the underlying causes of skin problems and provide targeted recommendations for anti-aging, moisturizing, sun protection, and other aspects. And recommend the suitable skincare ingredients or cosmetics, as well as diet, to achieve internal regulation and external nourishment.

### 5.2 Nutrition genetic testing

Nutrients such as vitamin A, vitamin D, Omega-3 fatty acids, etc. are closely related to skin health. These nutrients have a significant impact on the skin’s moisturizing, elasticity, antioxidant, and repair abilities. Individuals have differences in nutrient digestion, absorption, metabolism, and other processes, and the polymorphism of related genes can affect the activity of key enzymes.

Nutrition genetic testing provides users with three major nutritional requirement categaries: vitamin nutrition requirement, mineral nutrition requirement, and dietary metabolism. Specifically, it includes the testing items of: folate, vitamin A, vitamin C, vitamin D, vitamin E, vitamin K, vitamin B2, vitamin B3, vitamin B6, vitamin B12, iron, magnesium, selenium, zinc, calcium, phosphorus, lactose tolerance, alcohol metabolism, caffeine metabolism, Omega-3 intake requirements, sweet taste sensitivity, bitter taste sensitivity, etc.

Different nutrients use different nutrition requirement scoring algorithms, for example, folic acid requirement follows the rules from CDC (U.S. Centers for Disease Control and Prevention) (US Centers for Disease Control and Prevention, 2025), vitamin D requirement follows the polygenic risk score (PRS) (Jian et al., 2018).

**FIGURE 4:**
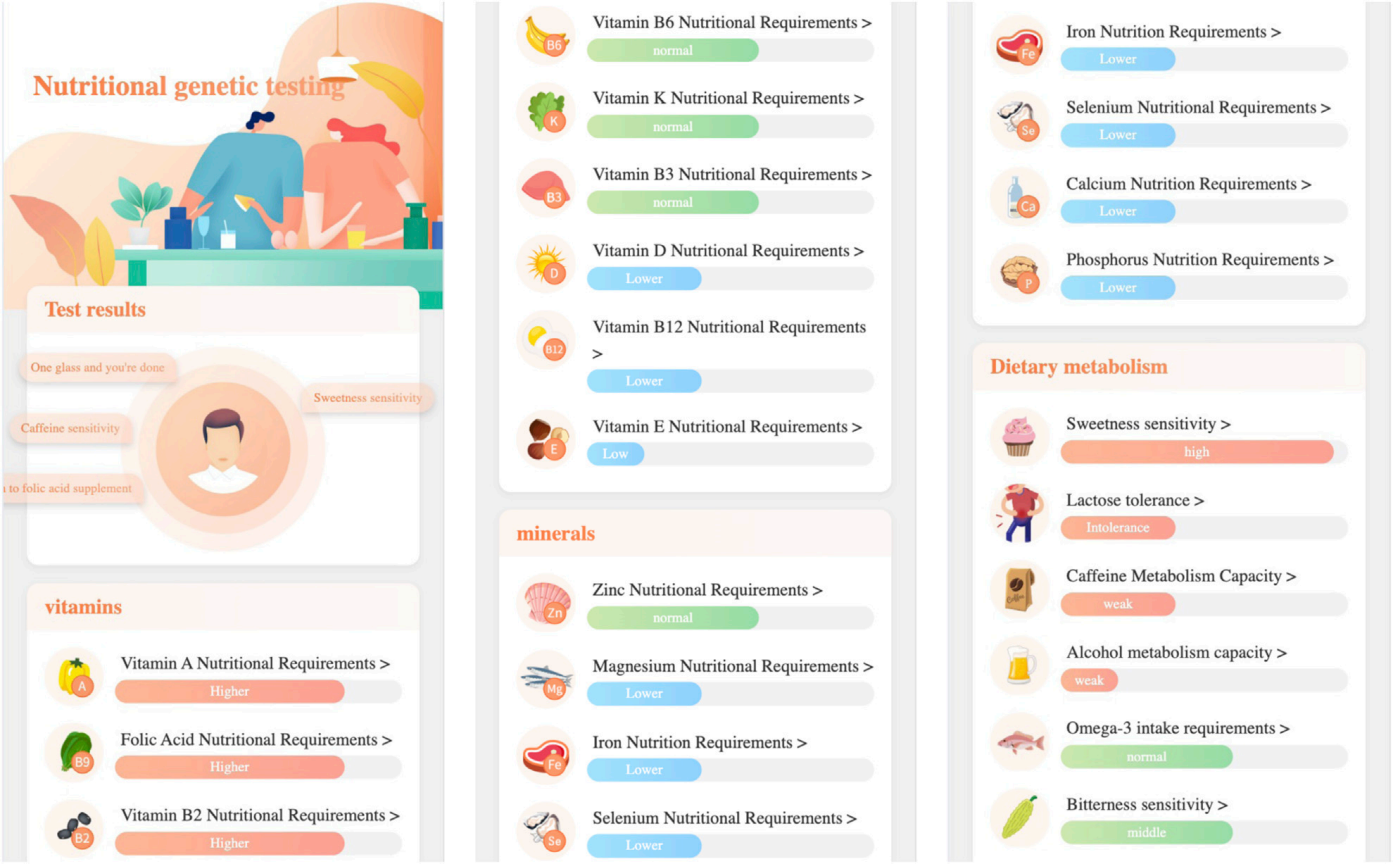
The nutrition genetic testing report’s first page.

Figure 4 gives the first page of nutrition genetic testing report, in which:

• The first module shows the testing items that need to pay attention;

• The second module lists the vitamin testing items (e.g., Vitamin A nutritional requirements) and their corresponding testing results (e.g., higher);

• The third module lists the mineral testing items (e.g., Zinc nutritional requirements) and their corresponding testing results (e.g., normal);

• The fourth module lists the dietary metabolism testing items (e.g., Sweetness sensitivity) and their corresponding testing results (e.g., high).

**FIGURE 5:**
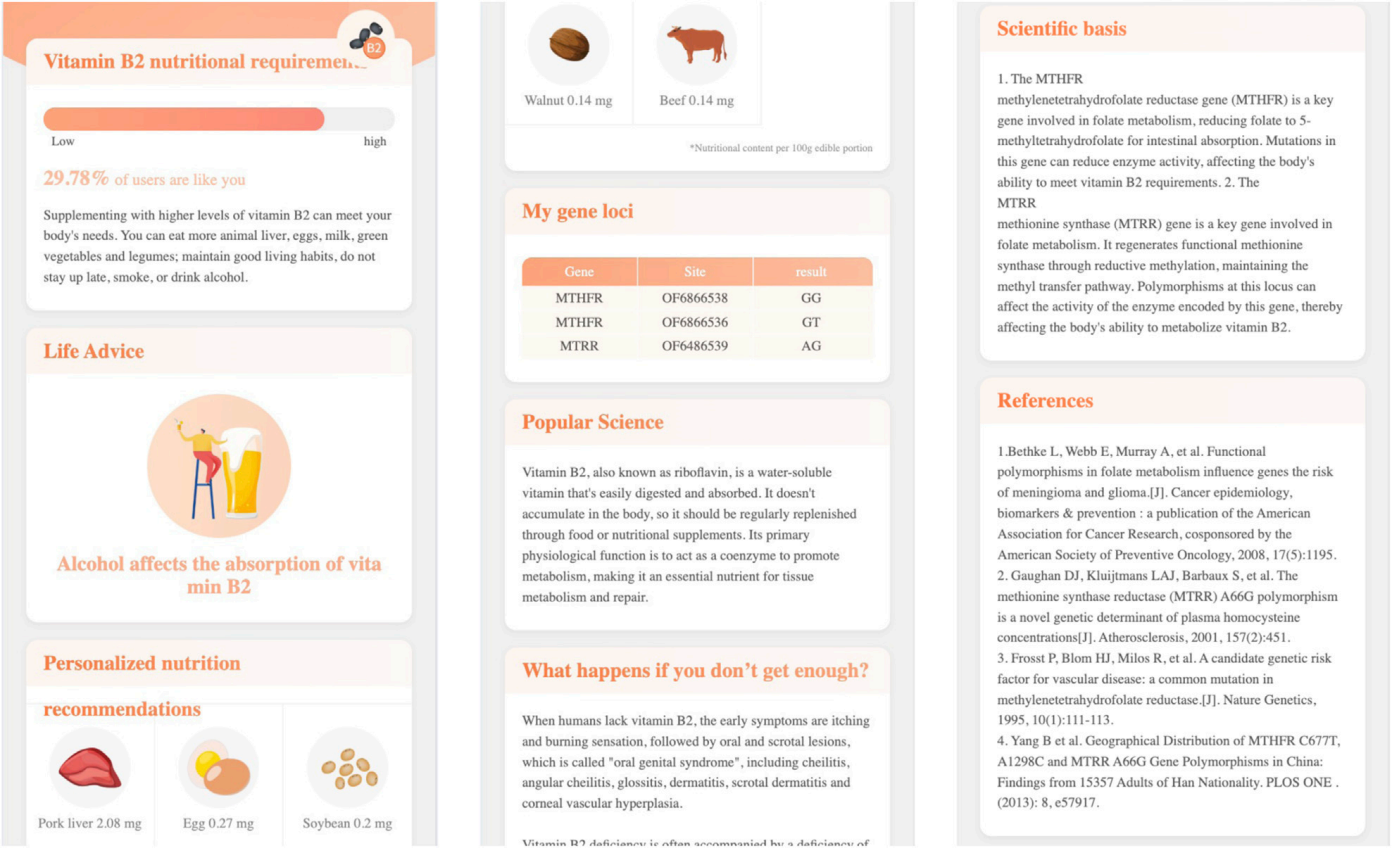
The nutrition genetic testing report’s second level page.

Click the name of each genetic testing item, the report page will open a second level page. For example, Figure 5 demonstrates the second level page of Vitamin B2 nutritional requirement, in which:

• The first module gives the testing result and the corresponding suggestions;

• The second module gives the lifestyle advice such as “Alcohol affects the absorption of vitamin B2”;

• The third module lists the foods containg vitamin B2, such as, pork liver contains 2.08 mg vatimin B2 per 100g edible portion;

• The fourth module lists the tested genes and loci;

• The fifth module gives the popular science that introduces the properties and functions of vitamin B2;

• The sixth module explains the importance of vitatmin B2 and describes the risk of vitamin B2 deficiency;

• The seventh module gives the scientific basis to explain the connections between vitamin B2 and genes;

• The eighth module lists the reference publications.

For the items with high risk of nutritional deficiency, high intake requirements, and weak metabolic capacity, the nutritional genetic testing report will provide specific dietary recommendations, daily advice, nutritional science popularization, and other targeted suggestions to accurately supplement nutritional needs, strengthen the skin’s nutritional foundation, and resist aging.

### 5.3 Lifestyle questionnaire

**FIGURE 6:**
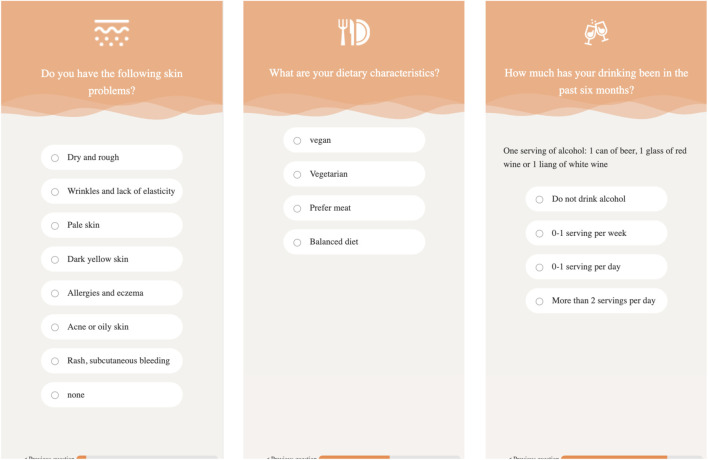
The Food Frequency Questionnaires pages.

In addition to the influence of innate factors on the skin, postnatal lifestyle and dietary habits also affect skin health. For example, excessive alcohol consumption can easily lead to dry skin, dilation of capillaries (risk of rosacea), consumption of vitamin B and zinc, and affect repair. Lack of sleep can lead to an increase in cortisol, damage to collagen, and increase inflammation; it can also lead to a decrease in melatonin, lower antioxidant capacity, and slow down skin repair (resulting in dull complexion and eye bags). Moderate exercise can promote blood circulation, transport nutrients, sweat and detoxify (but it needs to be cleaned in a timely manner); however, excessive exercise can lead to an increase in free radicals, which requires an antioxidant diet. Insufficient intake of antioxidant foods such as vitamin C (citrus, kiwi, broccoli) can easily lead to loose collagen fiber structure, decreased skin elasticity, wrinkles, and sagging. Therefore, we developed online food frequency questionnaires, as demonstrated in Figure 6, to understand user’s lifestyle and dietary habits. The questionnaires will cost 3–5 min to finish.

### 5.4 Personalized skincare nutrition solution

Skin is the second largest organ in the human body, and its health status is influenced by both genetic and environmental factors. Genetic testing can reveal individual genetic risks and provide a basis for developing personalized skin health management. Through skin genetic testing, individual’s various skin abilities can be understood; through nutrition genetic testing, individual’s nutritional metabolic risks and nutrition requriements can be understood. At the same time, by filling out the online lifestyle questionnaire, individual’s lifestyle and dietary habits can be understood. Combining genetic testing data and food frequencey questionnaires data, we can compute and provide personalized nutritional skincare solutions for users following different country’s dietary reference intakes (DRIs) (Office of Disease Prevention and Health Promotion, 2023).

**FIGURE 7:**
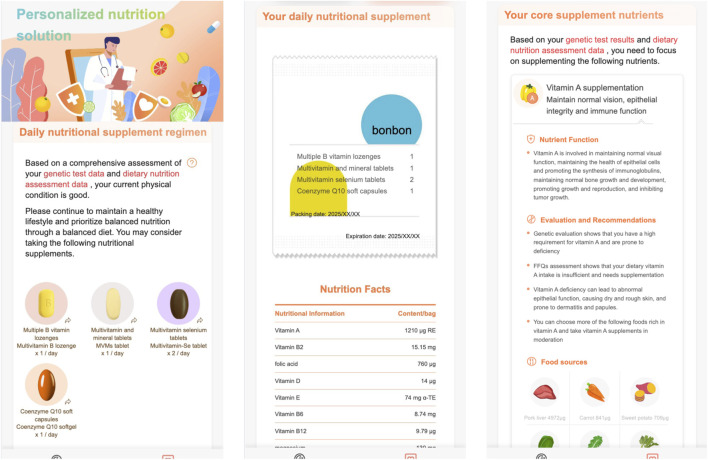
The personalized nutrition solution for skincare.

**FIGURE 8:**
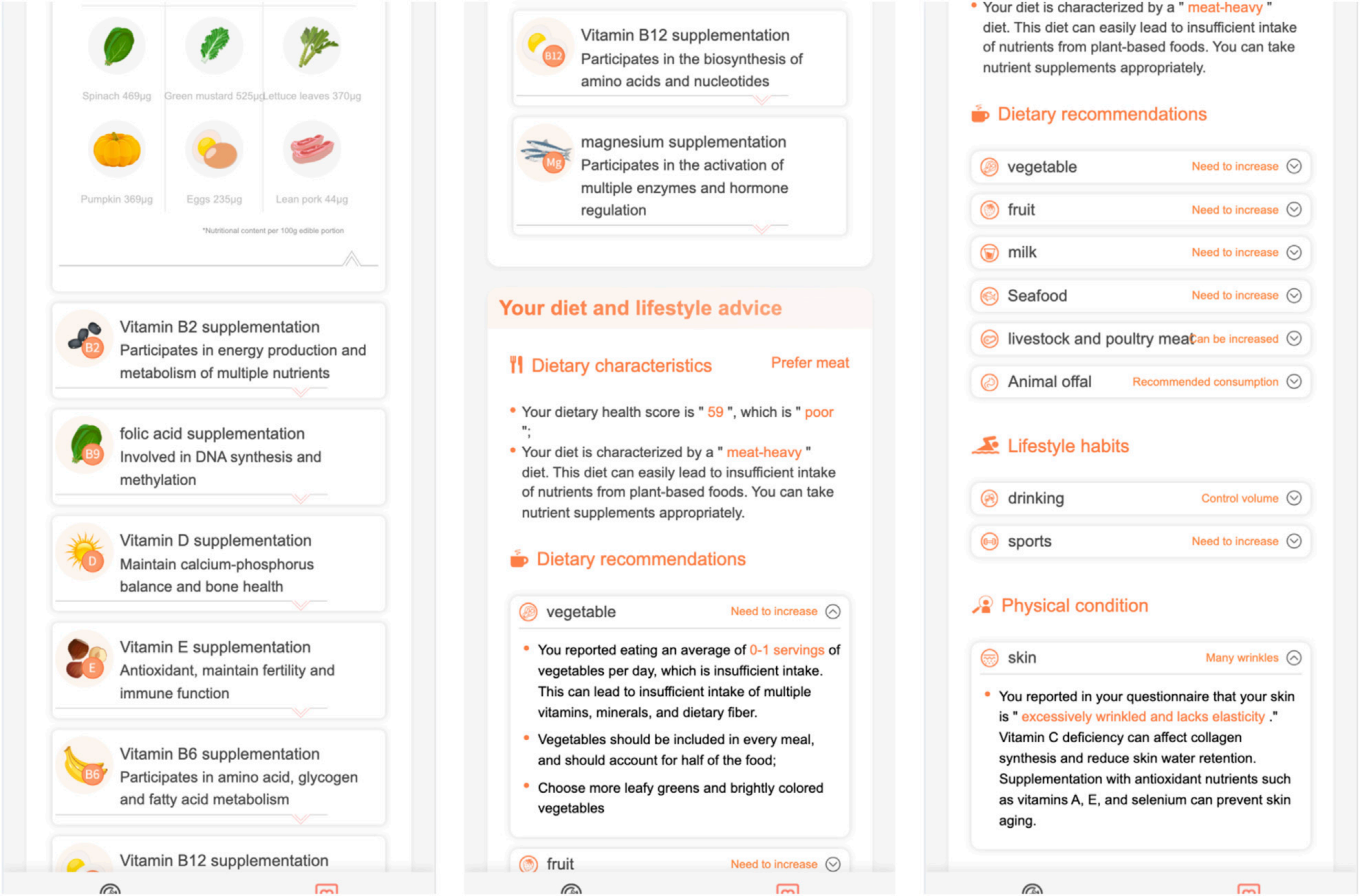
The personalized nutrition solution for skincare.

Figures 7, 8 demonstrate the personalized nutrition solution pages.

In Figure 7:

• The first page gives the personalized daily nutritional supplement solution which first explains that the personalized solution was generated based on the genetic testing results and food frequency questionnaires, then lists the customized supplements recommended for the customer;

• The second page simulates the appearance of a personalized nutrition pack, which includes the user’s name and a list of nutritional supplements in the pack;

• The third page lists the core nutritional ingredients recommended for the user to supplement, and provides the reasons why each nutrient is recommended, such as, for vitamin A supplementation,
  - it first summerizes the funcitons of vitamin A,
  - then the Nutrient Function section introduces the function of vitamin A in detail,
  - the Evaluation and Recommendations section explains why (i.e., based on genetic testing and food frequency questionnaires) vitamin A was recommended for the user,
  - the Food sources section lists the foods rich in vitamin A.

In Figure 8:

• The first page gives the other recommended nutrients for the user, and click the drop down icon on the nutrient name’s banner, more detail information will be shown like the third page of Figure 7;

• The second and third pages give diet and lifestyle advices,
  - the dietary characteristics section summerizes the user’s dietary habits,
  - the dietary advice section gives fruit, seafood, livestock and poultry meat suggestions,
  - the lifstyle section gives exercise advices,
  - the physical condition section gives skin care and hair care suggestions.

In order to evaluate the improvement effect of personalized nutrition on the skin, we used AI Skin Analysis (Zhou et al., 2024) technology to take photos and analyze the skin condition. By analyzing the skin condition before and after personalized nutrition intervention, we understood the skin condition and adjusted the formula of personalized nutrition as needed.

## 6 Conclusion

Precision skincare and personalized skin health management are cutting-edge fields that integrate dermatology, molecular biology, and nutrition. Their core concept is to deeply analyze the genetic basis and environmental interactions of individual skin characteristics through multi omics technologies such as genomics, metabolomics, and proteomics, in order to develop precise intervention strategies. In recent years, with the development of high-throughput sequencing and bioinformatics, researchers have identified numerous gene polymorphism sites associated with skin photoaging (such as MC1R, IRF4), pigmentation (such as TYR, SLC45A2), elasticity maintenance (such as COL1A1, ELN), and barrier function (such as FLG, SPINK5), and quantified an individual’s skin aging risk based on a polygenic risk score (PRS) model. These genetic markers not only reveal the molecular mechanisms of skin phenotype differences, but also provide scientific basis for the selection of targeted skincare ingredients, such as supplementing vitamin C/E or carotenoids for individuals with antioxidant deficiencies, or designing intervention plans rich in collagen peptides for those with abnormal collagen metabolism.

In terms of nutritional intervention, a large number of studies have shown that specific active ingredients (such as retinol, hyaluronic acid, polyphenolic compounds) can synergistically improve skin health through multiple pathways, such as regulating oxidative stress (such as inhibiting ROS generation), reducing inflammatory responses (such as modulating the NF - κ B pathway), and promoting extracellular matrix synthesis (such as activating TGF - β signaling). For example, vitamin A derivatives have been shown to significantly enhance collagen synthesis and reduce the degradation of matrix metalloproteinases (MMPs), while omega-3 fatty acids alleviate photoaging damage by repairing lipid barriers.

In this paper, we surveyed the genes related to skincare and proposed to integrate genes, nutrition, and lifestyle data to provide personalized skin care solution. The service has already been delivered and used by tens of thouands of customers.

Integrating multiple omics data and providing personalized solutions is expected to become one of the leading strategies for skin anti-aging and disease prevention, but more research and validation are needed in the future.

## Data Availability

The original contributions presented in the study are included in the article/supplementary material, further inquiries can be directed to the corresponding author.
